# Engagement of Fathers in Parent Group Interventions for Children with Congenital Zika Syndrome: A Qualitative Study

**DOI:** 10.3390/ijerph16203862

**Published:** 2019-10-12

**Authors:** Tracey Smythe, Antony Duttine, Ana Carolina Dias Vieira, Barbara da Silveira Madeira de Castro, Hannah Kuper

**Affiliations:** 1 The London School of Hygiene & Tropical Medicine, London WC1E7HT, UK; antony.duttine@lshtm.ac.uk (A.D.); hannah.kuper@lshtm.ac.uk (H.K.); 2 Pontificia Universidade Catolica do Rio de Janeiro, Rio de Janeiro 22451-900, Brazil; anadiasvieira@yahoo.com.br; 3 Instituto Fernandes Figueira, Rio de Janeiro 20021-140, Brazil; barbarasmcastro@gmail.com

**Keywords:** Zika, disability, parent support, low and middle income, father engagement

## Abstract

We aimed to explore the engagement of fathers in a community-based group intervention (Juntos) for children with congenital Zika syndrome (CZS) and their caregivers in Brazil. Six Juntos groups were facilitated from August 2017 to May 2018. We conducted a qualitative study to evaluate the feasibility and acceptability of the intervention for fathers of children with CZS. Methods included participant observation, focus group discussions, and semi-structured interviews of fathers with a child enrolled in the program. Data collected were transcribed, coded and thematically analyzed to explore father preference for, and beliefs about the intervention and to assess potential barriers and enablers to their involvement. Forty-nine families (61 participants) enrolled, of whom 20% (12/61) were fathers. Seven (58%) fathers attended more than 7 out of 10 sessions. The content of Juntos was found to be acceptable to those fathers who attended. Participation in the group offered fathers the opportunity to share experiences of caring for their child and demonstrate their importance as care agents. Work commitments, and the view of mothers as primary caregivers were barriers to engagement of fathers. Facilitators to engagement included a presentation of clear objectives for fathers’ involvement and the opportunity to learn a practical skill related to caring for their child. A better understanding of the perspectives of fathers is crucial to help increase their involvement in parenting interventions.

## 1. Introduction

There are an estimated 95 million children with disabilities, constituting one in 20 children globally (5.1%) [1]. Caregivers of children with disabilities experience pressure to meet the emotional and physical needs of their child, while at the same time maintaining family functioning [2]. Consequently, families of children with disabilities report social isolation, stigma, mental health challenges and increased financial and emotional strain [3,4,5]. The risk of fathers abandoning their child with disabilities is high [6], making families even more vulnerable.

Child development programs focus mostly on the role of the mother, as women are traditionally socially constructed as primary carers, and this minimizes the caring role of fathers in co-parenting [7]. Yet there is strong evidence that well-designed health interventions that include fathers positively impact on child development, wellbeing, and family functioning [8,9,10], particularly since social support has emerged as an important factor in influencing both parent and child functioning [11]. Despite this evidence, engaging with fathers is one of the least well-explored and articulated aspects of parenting interventions [12], and attendance at parent groups is much lower for men than for women [13]. Fathers are often recruited via women [14], and evaluation studies have been conducted mostly with mothers [15,16]. Insufficient attention is, therefore, given to reporting fathers’ participation and impact on child or family outcomes. There is little robust evidence as to “what works” [14,17] to engage fathers with parenting interventions, mostly restricted to families of children without disabilities [18]. Group interventions that successfully integrate fathers of children with developmental disabilities are rare.

One domain in which the engagement of fathers is relevant, but has not yet been explored is the care of children with Congenital Zika Syndrome (CZS). More than three thousand children have been born with CZS since the start of the Zika epidemic in Brazil, and children with CZS present with a range of neurological conditions and sensory and musculoskeletal impairments [19,20], secondary to central and perhaps peripheral nervous system damage. In describing CZS, Moore et al. [21] suggest five unique features:

- Severe microcephaly withpartially collapsed skull;
- Thin cerebral cortices with subcortical calcifications;
- Macular scarring and focal pigmentary retinal mottling;
- Congenital contractures; and
- Marked early hypertonia with symptoms of extrapyramidal involvement.

Nevertheless, a wider spectrum of developmental impairments may yet manifest in children who were exposed to Zika in utero as they continue their development [22].

Children with CZS are provided formal medical support services, which in Brazil are primarily delivered through tertiary care centers. However, innovative ways to provide support and education to families of children with CZS are required, given the wide-ranging and complex needs of children with CZS, and consequent strain on their families [23,24,25,26]. One such intervention is ‘Juntos’ (‘together’ in Portuguese), which is a facilitated participatory group intervention for caregivers of children with CZS that runs over 10 sessions [4,6]. Each Juntos group is facilitated by one ‘expert carer’, a mother of a child with CZS, and one allied health professional (physiotherapist, occupational therapist or speech therapist). Each session includes ice-breaker activities, practical sessions and group discussions, and lasts approximately 4 h. Topics covered include positioning and moving, eating and drinking, communication, play and early stimulation, everyday activities, community inclusion and disability rights (Table 1). An emotional support activity, as part of every session, is included to stimulate open and supportive discussion between caregivers about their successes and difficulties. The intervention aims to improve the quality of life of caregivers and children with CZS [27]. Program material is available from www.ubuntu-hub.org.

Within the context of the Juntos intervention we aimed to (i) explore fathers’ views about the program, and (ii) assess potential barriers and facilitators to fathers’ involvement in Juntos to support care for their child with CZS.

## 2. Materials and Methods

Ethical approval was gained from Instituto Fernandes Figueira—IFF/FIOCRUZ—RJ/MS 2.183.547 and LSHTM Ethics Ref: 13608. Informed consent was acquired from all participants. The study protocol was published in May 2019 [28].

Participants of the Juntos intervention were caregivers (mothers, fathers, grandparents or aunts) of children with neurologist-confirmed CZS in Rio de Janeiro and Salvador (Bahia), identified through clinical and therapy networks at the two sites. For the purposes of this study, we used CZS to describe any child with impairments that can be directly attributed to Zika. The impairments in children included either mild cognitive, communication or functional skill delay and ranged to severe delay in all three developmental categories. Identified participants were contacted by site coordinators about joining the groups, and a total of 49 children were recruited with a broad range of impairments. Six Juntos groups, each of 10 sessions, were run in Rio de Janeiro and Salvador (August 2017–May 2018) to pilot test and assess the feasibility of the intervention. All participants attending the intervention had been invited to participate in the feasibility study by the study site coordinator.

We conducted a qualitative study of the Juntos intervention between August 2017 and June 2018. The reasons for doing the research were explained, and data were collected by four female Brazil-based research assistants (psychologists). None of the psychologists worked in the clinical area or had prior knowledge of the participants. The number of program sessions attended between baseline and program completion were recorded for each participant, and three techniques were used for collecting qualitative data. First, participant observation by a psychologist was undertaken during the delivery of all the sessions (*n* = 10 sessions) of the Juntos intervention in the six groups. An observation checklist was used to assess the fidelity of delivery of the intervention by the facilitators. In addition, observation of the ice-breaker activities, practical sessions and group discussions provided an opportunity to examine the interaction of the fathers with their child and their partners during each session. The psychologists observing the sessions wrote detailed field-notes on individual men’s engagement. Second, the psychologists (one per site) facilitated focus group discussions related to the content of the session at the end of each session. Focus group discussions were approximately forty-five minutes in duration. The psychologists took comprehensive notes of the discussion with the participants. For conversations which took place with facilitators outside of the sessions, pertinent comments were recorded in an excel file. Third, one-to-one semi-structured interviews were undertaken by the psychologists with caregivers post-intervention. All participants who had attended the Juntos groups were eligible for selection, and then a subsample of caregivers (*n* = 13) out of those who expressed an interest in being interviewed were purposively selected to include male and female caregivers and participants of different ages to provide a diverse sample. The 13 participants had completed between 7 and 10 sessions. The interview guide was adapted from a previous study [6], and piloted for understanding. Interviews were designed to explore participants’ motivation for attending the intervention, their views around how they were approached to participate in ‘Juntos’, how they felt about the program itself, and aspects around engagement were explored. The interview guide is provided as Supplementary 1. Interviews lasted on average 1 h, ranging between 50 min and 1 h 20 min and were recorded through audio techniques.

Audio-recorded data were transcribed verbatim and translated into English. The responses to the direct observation data and open-ended questions were collated into a word document by one lead UK based researcher, a specialized pediatric physiotherapist trained in qualitative and quantitative techniques (TS). The transcribed text was analyzed using an inductive thematic analysis [29]. This included three steps. First, two psychologists (A.C.D.V. and B.S.M.) who collected and transcribed the data, and who were, therefore, familiar with the content, read the text several times to form an impression of the overall content. Second, words and phrases that described fathers’ views of the intervention and that identified barriers and facilitators in the transcripts by both psychologists and were checked and verified by TS. These words provided an initial coding framework, and the preliminary codes were assigned to the data to describe the content. Third, the codes were collated into potential themes that were reviewed and refined. We undertook a narrative description of themes raised in the participant questionnaires, focus group discussions, interviews and conversations with facilitators. Consensus on emergent themes was reached through regular discussions. The themes were defined and named following agreement. Illustrative quotes are presented in the findings. We reported the results according to the consolidated criteria for reporting qualitative research (COREQ) [30], which is a 32-item checklist for interviews and focus group (Supplementary 2). Findings were not presented to the participants, and they were not invited to give feedback on the findings.

## 3. Results

Altogether, 49 families (61 participants) enrolled and attended at least one session of a Juntos group. Of these, 20% (12/61) were fathers, and 58% (7/12) fathers attended more than seven sessions. One father was parenting his child on his own, and all other fathers attended the groups with their partners. The four psychologists spent over 240 h in the facilitated group sessions, observing, and discussing with the participants. Sixty-two focus group discussions were held.

Through observation, the psychologists perceived that fathers initially participated in the group as a guide for their child and female caregiver.

“The fathers arrive to accompany the mothers in the group and it does not seem easy for them to feel comfortable talking about their feelings and difficulties with parenting.”

(Psychologist 01, Rio de Janeiro)

The ice-breakers and practical activities, such as practicing feeding each other, helped the fathers to engage with the group. The facilitators of the group had an important role in encouraging fathers to contribute and providing space for the fathers to talk and the psychologists observed:

“When they [fathers] are encouraged and comfortable in the group, they bring up very important topics and are able to show great sensitivity to parenting (…) they demonstrate to mothers that they [fathers] can be good caregivers and that mothers need not be the only specialists in caring for their children.”

(Psychologist 01, Rio de Janeiro)

Themes that emerged from the analysis were organized into: (1) Fathers’ preference for, and belief about, the Juntos group intervention; and (2) Barriers and enablers to fathers’ engagement in the Juntos intervention.

### 3.1. Fathers’ Preference and Beliefs

#### 3.1.1. Advantages of Group Format

Participation in the group offered fathers the opportunity to share similar experiences in caring for their child with complex multiple needs and fathers talked about the benefits of this:

“I see the power of this group, this is one of the most important things. It is good to know that there are others in the same situation as you; it gives you a greater strength.”

Father 03, Rio de Janeiro

“I am moved to hear someone else talking about difficult moments at her child’s birthday, when she felt people looking at her child with prejudice (…) She cried, and I felt the same feeling I’ve already felt so many times with my own child.”

Father 05, Salvador

The group setting also provided an environment in which to speak and listen to others in similar situations. The face-to-face format was viewed by fathers as acceptable, due to the benefits of social relationships and learning from others. Fathers were motivated to participate when they were offered speaking space within the parent group.

“It is important to be able to speak and know that someone else is available to listen. It is good to express some feelings that are almost physiological.”

Father 01, Salvador.

When fathers were offered speaking space, they voiced knowledge of their children, concerns about the reality in which they live and social challenges.

#### 3.1.2. Importance of Clear Objectives and Goals

Fathers attending the Juntos group suggested that including clear goals for their involvement was useful, but had not always been provided before the start of the group. Fathers reported not being aware of the aim of the program and what topics would be covered.

“I [initially] thought the aim of the program was to help mothers manage in a better way, because it really is not easy. But the day to day logistics are complicated for both parents, and family members.”

Father 02, Rio de Janeiro

“I thought this was going to be an academic study like the others, which didn’t influence anything in our lives (…) but when we came to the group, my first impression was the welcome we received from the technical team. They were very kind, affectionate, very polite and they explained the project (…) and that encouraged us to attend and to participate.”

Father 05, Salvador

#### 3.1.3. Practical Learning through the Intervention

The content of Juntos was found to be acceptable to fathers who attended the groups. For example, both mothers and fathers expressed appreciation of the group format, but there was a difference between parents in the perceived value of the information taught. Fathers saw themselves as being engaged predominantly in their child’s care through practical and resource support. Fathers, in comparison to mothers, requested pictures and information that could be applied, such as how to brush their child’s teeth in a practical way. They also suggested the use of more stories and repeatedly asked for case studies, photographs and videos of children that were older:

“Sometimes the positions that are suggested to be better for my child are not comfortable for her. So, what do I do? I would like to see how older children progress.”

Father 05, Salvador

This feedback from fathers during the first phase was used to reform the content of the program; case studies, videos of fathers helping their child with everyday activities (such as brushing teeth and playing in a park) and images of fathers building supportive equipment were developed for the intervention.

Fathers demonstrated initial hesitation with participating in some activities in the program, perhaps to a greater extent than mothers, although these were often overcome:

“At the beginning it was a bit weird, funny, and I was shy (…) then I saw the activity and I was involved, and I had fun.”

Father 05, Salvador

“Even if you have more difficulty, if you are shy (…) the activities are good so that everyone does not just sit and be quiet.”

Father 02, Rio de Janeiro

#### 3.1.4. Improved Knowledge and Skills

Fathers viewed improved knowledge and skills as a way to demonstrate their importance as care agents for their child and family. Positive outcomes of participating in the group reported from the fathers’ perspective included increased communication with their child, and learning a practical skill that was helpful for the care of their child, such as creating supportive equipment:

“Right after the communication module I started to talk more with my daughter. I talk with her when we are playing together, and she answers me laughing.”

Father 07, Salvador.

“After the positioning module I adapted a shower chair. We take this little chair everywhere we go (…) because she opens her eyes more when she is seated. That it is why I take the chair. She keeps looking, it increases her curiosity.”

Father 06, Salvador.

All of the interviewed fathers reported that the parent group helped them to understand their child’s development and improved their confidence in caring for their child. Fathers viewed several topics relevant to child development as important for understanding their role as a father in caring for a child with complex needs, including Session 3: Positioning and Moving, Session 5: Communication, and Session 6: Play and Early Stimulation.

“I liked the positioning and moving session. We learned with each other and shared tips, and now I know how to help my child to progress and develop through play.”

Father 01, Salvador.

“We learned about equipment. I understand now that she needs a tray to support her arms in a standing frame. I can help her to do this.”

Father 06, Salvador.

### 3.2. Barriers and Facilitators to Engagement

#### 3.2.1. Time of Delivery

Groups in Salvador were held on Saturdays and groups in Rio de Janeiro were held during the week. The timing and place of program delivery proved a barrier to fathers’ attendance, as they were often at work, which was less of a concern for mothers’ attendance.

“But maybe on the weekend, Saturday, Sunday, could be a solution because during the week the schedule is hard. I try to be present.”

Father 02, Rio de Janeiro.

#### 3.2.2. Cultural Norms about Fatherhood and Marital/Partner Relationship

In general, mothers identified with the role of primary carer for everyday need. Fathers that participated in the group were observed to demonstrate affection for their child and voiced desire for a better quality of life for their child. However, women attending the groups perceived that the contribution of men in childcare needs to be increased. This issue was not raised by any of the fathers, and the barrier may be driven by the marital/partner relationship.

“And there it is: [T]he mother leads all the activities with the child, who comes to the group? The mother. The person that practices with the child is the mother. I think it is a cultural thing. But if we had some way (…) to bring the father (…) some formal invitation or something written for him so he can see that he is important too.”

Mother 12, Rio de Janeiro

“I learned that I have to talk to him [husband] (…) and I sat down with him to talk about a consultation (…) and he said: I like to know things, but you do not like to speak. In my head it was he [husband] who was not interested.”

Mother 14, Rio de Janeiro

The invitation to join the group was administered to the mother, who then became the gate-keeper as to whether the father was invited and encouraged to attend. Whilst women in the group also stated that having fathers there was a positive experience, this was contradicted by other mothers who wanted space to be able to raise issues about the burden of caregiving in a protected space without the father:

“We want some space without our husbands, so we can relax, we can talk about them, about a husband who pushes his wife all the time. My husband says ‘our son is not getting any better, because you don’t do the exercises properly.’ When I go home I have to do so many things, prepare meals, cleaning (…) how could I do more exercises?”

Mother 11, Rio de Janeiro.

## 4. Discussion

We explored fathers’ preferences for, and perceived barriers to, a group intervention to support care for children with CZS. The group format and content of Juntos was found to be acceptable to fathers who attended the groups. During the development of the content for the Juntos intervention, feedback from fathers during the first phase was directly utilized in the reforming of the program (for example providing case studies, images and films of fathers undertaking practical tasks), which may have made the overall content more useful and acceptable for the later groups. Involving men in program design and implementation is an important factor for promoting male involvement in programs [26]. The fathers who attended over seven of the sessions reported changes in behavior and confidence in caring for their child. The main perceived benefits of the program by fathers were the opportunity to share experiences of caring for their child, and the chance to demonstrate their importance as care agents. Fathers saw themselves as being predominantly engaged in their child’s care through practical and resource support.

There remain fewer fathers engaged than mothers as only one in five of the participants of the groups were fathers. However, children with CZS in Brazil may have had more fathers involved than with other similar related conditions, such as cerebral palsy, as expressed by several members of medical and rehabilitation teams [28] from the initial scoping visit for Juntos in April 2017. One possible reason identified was that the high level of discussion and awareness-raising through the media on Zika has meant that there was much less shame related to having a child with CZS than with other similar conditions. Fathers were limited by availability as they saw themselves as the provider of financial support; taking time from work was a barrier to attendance. Women attending the groups perceived that the contribution of men in childcare was not equal, and saw the need for this to change. However, not all women were supportive of fathers being included in parenting interventions.

The difficulty in engaging fathers with parenting programs needs to be addressed given the positive impact that fathers have on their child’s behavior and development (e.g., school readiness, cognitive development and pro-social behaviors) [14], and the frequency of paternal abandonment of disabled children [5]. In addition, when both parents engage in parenting programs, the outcomes for children are more positive [31]. A meta-analysis found that, compared to mothers, fathers have a greater ability to influence a child’s misbehaviors [32], and promotion of effective co-parenting enhances family functioning and child outcomes [33], but evidence on these father-related outcomes for children with disabilities is lacking [34,35].

These findings have important implications for the targeting and tailoring of parenting interventions in order to increase father engagement, including the need to (i) identify and support strategies to involve fathers from the beginning of interventions, (ii) clearly communicate the goals of the group and what can be expected to be achieved, (iii) provide opportunities for both fathers and mothers to participate, (iv) develop media that shares the men’s point of view and includes illustrations of fathers, and (v) include practical information as requested by fathers. It must be ensured that perceptions of childcare as “women’s work” are not reinforced. In addition, strengthening fathers’ involvement also requires increased awareness from mothers about the need and importance of care offered by fathers to their children.

Alternatives to regular participation in group interventions are also required, since men may be unable to attend the group, do not see its importance, or do not want to engage in group activities or discussions, or mothers may want to have a group consisting solely of women. Alternatives include the development of more printed material, a detailed website, and a greater focus on practical information.

The design of this study has strengths and limitations that need to be considered. This study used qualitative methods to evaluate the engagement of fathers in a group intervention to improve the quality of life of children with CZS and their caregivers. Data presented provides multiple perspectives, from both fathers and mothers, which is a strength of this study. Limitations include the inability to interview any fathers that did not take part in the group intervention about reasons for non-attendance, and the small number of fathers included and interviewed. In addition, participants included some fathers who had not previously participated in group interventions, which may increase the applicability of the findings to fathers who have little experience of parenting interventions. However, we did not specifically measure how many fathers had previously participated in parenting interventions, so we are unable to quantify this. Generalizability of the findings may be limited. Factors that differentiated those that completed the program compared to those that did not were, therefore, not explored. The use of psychologists (non-clinicians) as researchers has implications for assessment of clinical data; however, the use of a specialized pediatric physiotherapist as the third researcher reduces the likelihood of inaccuracies in interpretation of these data. With regards range of impairment in children, we could not compare the severity of impairment of those who participated versus those who did not.

## 5. Conclusions

The group format and content of Juntos was found to be acceptable to those fathers who attended. Alternatives to regular participation in parent group interventions may be required to provide support and education to fathers of children with CZS. Perspectives, needs and preferences of fathers within their context should be considered in the design and delivery of parent group interventions.

## Figures and Tables

**Table 1:** Juntos intervention module topics.

Module Number and Title	Topics Covered
1: Introduction	About the program Information about Zika and Congenital Zika syndrome How to find information Personal stories
2: Our child	Introducing your close family and friends Development milestones for young children Determining your child’s progress Managing irritability and crying
3: Positioning and moving	How to position children who need assistance How to assist children to learn to move
4: Eating and drinking	Feeding challenges Practical skills to address challenges for your child
5: Communication	Importance of communication Practical advice to help your child communicate
6: Play and early stimulation	Importance of play for children to develop and learn Early stimulation Making simple toys Inclusion of play in the family and the broader community
7: Everyday activities	How to use everyday activities to help your child develop Managing seizures
8: Uniting our voices	Understand the context of disability rights Education Communicating with your health team Advocating
9: Our community	Who is in your community Common barriers to inclusion Addressing negative attitudes and exclusion Social Activity
10: Next steps	Summing up Planning next steps for yourself and the group

## Supplementary Materials

The following are available online at https://www.mdpi.com/1660-4601/16/20/3862/s1, Supplementary 1: COREQ checklist, Supplementary 2: Topic guide: Post-intervention participant interviews.
